# Synaptic Learning Rules and Sparse Coding in a Model Sensory System

**DOI:** 10.1371/journal.pcbi.1000062

**Published:** 2008-04-18

**Authors:** Luca A. Finelli, Seth Haney, Maxim Bazhenov, Mark Stopfer, Terrence J. Sejnowski

**Affiliations:** 1 Computational Neurobiology Laboratory, The Salk Institute for Biological Studies, La Jolla, California, United States of America; 2 Howard Hughes Medical Institute, The Salk Institute for Biological Studies, La Jolla, California, United States of America; 3 National Institutes of Health, NICHD, Bethesda, Maryland, United States of America; 4 Division of Biological Sciences, University of California San Diego, La Jolla, California, United States of America; UFR Biomédicale de l'Université René Descartes, France

## Abstract

Neural circuits exploit numerous strategies for encoding information. Although the functional significance of individual coding mechanisms has been investigated, ways in which multiple mechanisms interact and integrate are not well understood. The locust olfactory system, in which dense, transiently synchronized spike trains across ensembles of antenna lobe (AL) neurons are transformed into a sparse representation in the mushroom body (MB; a region associated with memory), provides a well-studied preparation for investigating the interaction of multiple coding mechanisms. Recordings made *in vivo* from the insect MB demonstrated highly specific responses to odors in Kenyon cells (KCs). Typically, only a few KCs from the recorded population of neurons responded reliably when a specific odor was presented. Different odors induced responses in different KCs. Here, we explored with a biologically plausible model the possibility that a form of plasticity may control and tune synaptic weights of inputs to the mushroom body to ensure the specificity of KCs' responses to familiar or meaningful odors. We found that plasticity at the synapses between the AL and the MB efficiently regulated the delicate tuning necessary to selectively filter the intense AL oscillatory output and condense it to a sparse representation in the MB. Activity-dependent plasticity drove the observed specificity, reliability, and expected persistence of odor representations, suggesting a role for plasticity in information processing and making a testable prediction about synaptic plasticity at AL-MB synapses.

## Author Summary

The way in which the brain encodes, processes, transforms, and stores sensory information is a fundamental question in systems neuroscience. One challenge is to understand how neural oscillations, synchrony, population coding, and sparseness interact in the process of transforming and transferring information. Another question is how synaptic plasticity, the ability of synapses to change their strength, interacts efficiently with these different coding strategies to support learning and information storage. We approached these questions, rarely accessible to direct experimental investigation, in the olfactory system of the locust, a well-studied example. Here, the neurons in the antennal lobe carry neural representations of odor identity using dense, spatially distributed, oscillatory synchronized patterns of neural activity. Odor information cannot be interpreted by considering their activity independently. On the contrary, in the mushroom body—the next processing region, involved in the storage and retrieval of olfactory memories and analogous to the olfactory cortex—odor representations are sparse and carried by more selective neurons. Sparse information coding by ensembles of neurons provides several important advantages including high memory capacity, low overlap between stored objects, and easy information retrieval. How is this sparseness achieved? Here, with a rigorous computational model of the olfactory system, we demonstrate that plasticity at the input afferents to the mushroom body can efficiently mediate the delicate tuning necessary to selectively filter intense sensory input, condensing it to the sparse responses observed in the mushroom body. Our results suggest a general mechanism for plasticity-enabled sparse representations in other sensory systems, such as the visual system. Overall, we illustrate a potential central role for plasticity in the transfer of information across different coding strategies within neural systems.

## Introduction

Neuronal circuits implement a variety of coding strategies that differ in their reliance on precise timing and correlations between action potentials (“spikes”). Among these strategies are oscillations, synchronization and precise spike timing on the one end of the spectrum; population codes and firing rate changes on the other one [1]–[6]. The functional significance of these distinct coding strategies has been investigated in different sensory modalities, yet their interaction and integration within neural systems remains an open question in the theory of neural coding. One reason for this is that it is generally not possible to make direct and precise experimental observations of the interplay between information flow and neural circuitry at different levels of sensory processing.

A notable exception is provided by the invertebrate olfactory system, where the relationship between oscillatory synchronization and sparse codes, the role of neuronal plasticity, coincidence detection and oscillatory synchronization have been investigated both experimentally [6]–[8] and with realistic computational models [8]–[11].

The oscillatory activity reflected in electroencephalogram (EEG) and local field potential (LFP) recordings has been associated with sensory processing [1]–[3],[12] and cognitive states in humans [13]–[15]. The biophysical and network mechanisms underlying oscillatory activity in the brain have been well characterized, and involve periodic coherent synchronization of neuronal assemblies [3],[10],[16],[17]. In particular, in the antenna lobe (AL) of the locust [3] (the analog of the vertebrate olfactory bulb), oscillatory synchronization is a feature of population coding. During the presentation of an odorant, transiently synchronized, evolving ensembles of neurons are formed and together participate in the encoding of the odor [3],[10],[11].

The significance of the AL's odor oscillatory coding scheme can be best understood by examining postsynaptic units that read the oscillatory output. In insects, these follower units include neurons in the mushroom body (MB), a structure known to be involved in the storage and retrieval of memory representations [18]–[20]. In locusts, although a large fraction of the 830 AL projection neurons (PNs) participates in a dense and dynamic oscillatory response, only a very small subset of the 50,000 Kenyon cells (KCs) in the ipsilateral MB actively responds to any single odor [7]. Individual KC responses are rare and consist, on average, of only a few action potentials. Thus activity in the MB is sparse [7], and population codes mediating odor representations differ markedly between AL and MB. Similarly, studies in mice have shown that individual odorants are represented by subsets of sparsely distributed cortical neurons [21].

The transformation of odor responses from AL to MB requires the tendency of PNs to synchronize through oscillatory dynamics, the ability of KCs to respond as coincidence detectors, activated only by correlated input from the AL [7],[8], and a precisely and actively tuned match between coding and decoding processes. What mechanisms underlie this match and enable the appropriate selection of coherent oscillatory signals, fundamental for reading the information contained across synchronized PN spike trains?

A form of activity-dependent plasticity between AL and MB may provide the necessary fine-tuning for ensemble selection in the MB, as well as a substrate to shape persistent sparse odorant representations. We explored this hypothesis here with a biologically plausible computational model. This model includes the main processing steps of the insect olfactory system, but was designed to be general enough to allow comparisons to vertebrate olfaction and to sensory processing in other brain areas. Two different forms of dynamic regulation of synaptic strength were compared, one based on the precise millisecond timing of individual spikes [22]–[24] and a second that depended on the rate of spike occurrence averaged over longer intervals [25],[26]. We found that plasticity can efficiently tune the selectivity for synchronized input, generating sparse, reliable and persistent representations to familiar or meaningful odors in the MB. Finally, we found the model makes an experimentally testable prediction about plasticity mechanisms governing synapses to KCs. Although this model is focused on the locust olfactory system [7], electrophysiological recordings and theoretical considerations suggest that sparseness could be a ubiquitous coding strategy exploited by several different modalities across different organisms (reviewed in [5]). Thus, this relatively simple system constitutes a favorable model for studying the interplay of neural codes with plastic mechanisms of learning and memory.

## Results

### Synaptic Plasticity in the Mushroom Body

**Figure 1:**
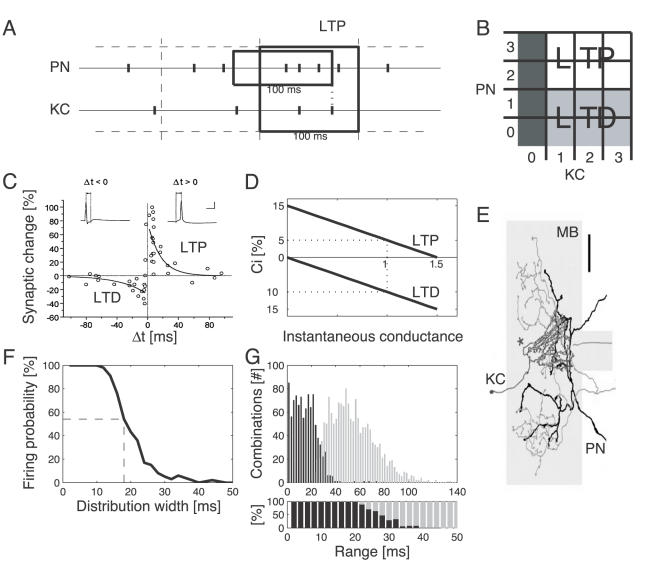
Synaptic plasticity and coincidence detection in the model Kenyon cell. (A) The SRDP rule was applied every 100 ms (large box over dashed time grid, see Materials and Methods). For each KC spike within that window, the instantaneous PN firing rate was estimated in the preceding 100 ms (small box; for this potentiation example: 3 spikes, or 30 Hz). (B) SRDP learning rule look-up table for potentiation (white), depression (light gray), and no change (dark grey), embodying the interaction of a PN discrete covariance term and a KC binary term. Axes indicate spike counts in 100 ms windows, as exemplified in A. (C) The pairing time window for STDP was determined by two exponentially decaying functions critically depending on the timing difference between the onset of the excitatory postsynaptic potential (EPSP) and the peak of the postsynaptic action potential. (See Materials and Methods; from [57]. Copyright 1998 by the Society for Neuroscience. Reprinted with permission.) (D) Linear dependence of synaptic change on instantaneous synaptic strength (factor Ci, Equation 1). Values normalized by the initial conductance g*. (E) Representative connection between a PN axon (black) and a KC dendritic tree (gray) in the MB calyx (light gray). (From [7]. Reprinted with permission from AAAS.) (F) Gaussian distributions of increasing width ( = 2×STD, abscissa) generated input trains of 10 spikes with decreasing probability of triggering a KC spike (see also [G]). Probability was higher than 50% for widths of 18 ms or less (dashed line). (G) Input combinations generated in (F) resorted according to the actual interval between first and last spike (range). Coincidence detection events (KC spike; black bars, 2-ms steps) predominated against non-firing combinations (gray bars) for ranges up to 26 ms (upper panel: total count; lower panel: relative count). Coincident spikes within a 20 ms range were always detected.

In insects, projection neurons (PNs) of the antennal lobe (AL) are the only source of olfactory information to the mushroom body (MB). We focused on the effects of plasticity at the synapses connecting PNs to Kenyon cells (KCs) in the MB calyx (Figure 1). Each synapse was characterized by a nonnegative peak conductance *g*, and synaptic plasticity was realized as a change in *g* by the amount *Δg*, proportional to three independent factors:

1. *L_r_* ^±^: the learning rule factor
2. *C_i_*: linear function of instantaneous synaptic conductance
3. *F_p_*: binary function of pairing frequency
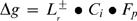
(1)

Learning rules based on spike rate- or spike timing-dependent induction of synaptic plasticity are central in influential models of cortical learning and neural development [22]–[26]. We investigated the contributions of rate- and timing-based mechanisms in the MB with a series of computational experiments. Specifically, we compared spike-timing dependent plasticity (STDP; Figure 1C) with a form of plasticity that depends on the rate, but not precise timing, of pre- and postsynaptic firing (Figure 1A and 1B). By analogy with STDP [27], we termed this spike-rate dependent plasticity (SRDP).

**Figure 2:**
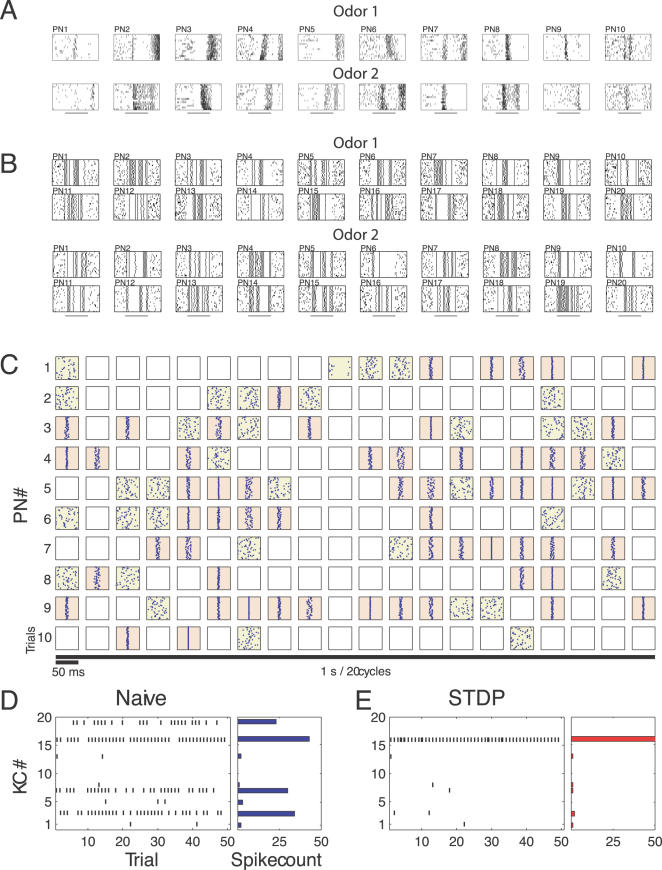
AL and MB population codes. (A) Firing patterns of 10 PNs for 2 different odors *in vivo*. Each odor (horizontal bars, 1 s) was presented 15 times (raster plot). (B) Firing patterns of 20 modeled PNs are shown for two different odors. Each box represents activity for one PN. Each stimulus (horizontal bars, 1 s) was presented 20 times and included small variations between trials. Approximately one-half of the inputs were synchronized at each oscillation cycle, and identities of the synchronized inputs changed slowly over the stimulus duration (see Materials and Methods). (C) Typical AL timing structure; input spike trains to one KC from 10 PNs (rows) during 1-s odor presentation (1 block of stimuli: 50 trials with the same odor). Consecutive boxes on each row represent the spike raster of a PN for each cycle (50 ms) of the 20 Hz LFP oscillation. Pink boxes indicate PN-cycle combinations of reliable synchronous spiking across trials (aligned spikes; STD<5 ms); i.e. the instantaneous PN ensemble exhibiting oscillatory synchronization in the corresponding cycle. Yellow boxes indicate PN-cycle combinations with spiking activity without synchrony. Empty boxes indicate silent cycles. Synchronized PNs may be assumed phase-locked to the positive peak of the LFP oscillation. (D) Quantification of KC population response before learning (naive). Spiking activity of all 19 KCs (left panel) during 50 presentation trials of the same odor. The corresponding spike count bar plot is shown in the right panel. (E) Quantification of KC population response after learning with STDP. Note the increasing level of sparseness in the KC population. Procedure as in (D).

In most of our computational experiments, we compared the effects of SRDP and STDP at the PNs-to-KCs synapses (Figure 1E). For each experiment, the population response in KCs was quantified before (“naive” case; e.g., Figure 2D) and after training (e.g., after STDP: Figure 2E) over repeated presentations of blocks of olfactory stimuli (see Materials and Methods).

Our simulations were based on small populations of conductance-based KCs receiving input from 100 PNs (i.e., ∼12.5% of total locust PN population). Each model KC received monosynaptic input from 10 PNs [7]. To reduce the likelihood of facilitating sparseness by independent connectivity, pairs of KCs shared 50% of their input afferents [8]; an extra overlap that did not otherwise affect the results. Following an approach that we previously applied in [8] the PN activity of the model (Figure 2B; see Materials and Methods) included dynamic ensembles of synchronized neurons replicating the behavior of PNs recorded *in vivo* (Figure 2A) [7],[28],[29] as well as of PNs in a complete AL model [9],[10].

### Synaptic Plasticity Enhances Sparseness and Selectivity of Olfactory Representations in KCs

**Figure 3:**
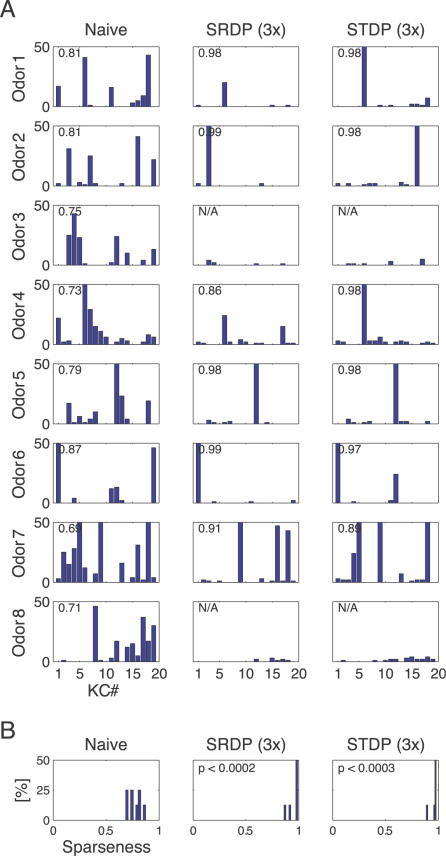
Synaptic plasticity favors sparse representations in the MB. (A) Responses of the KC population to 8 different odors before and after synaptic plasticity. Left column: spike count bar plots for naive responses. Middle column: responses after SRDP-based synaptic tuning during presentation of 3 blocks of olfactory stimuli. Right column: the same for STDP. The sparseness measure *Sp* for each condition is indicated in the upper left corner of the bar plots. (B) Histogram of the sparseness measure across the 8 different odors, in the naive, SRDP, and STDP cases. Note the significant increase in sparseness after introduction of both synaptic plasticity mechanisms, compared to the naive system (paired *t*-test).

Before olfactory experience, stimulus-evoked response patterns in the MB were dense, with olfactory representations characterized by the participation of a large fraction of the available KCs (often >25%; Figure 3A, left column). Most neurons fired less than one spike per trial (see spike raster in Figure 2D); thus, reliability was low. Population responses were unselective and largely overlapping across different odors (Figure 3A, left column). Both learning mechanisms made KCs population responses sparser (Figure 3A, middle and right column), such that in most cases only one KC became specifically responsive to a given odor. After olfactory experience with SRDP or STDP over 3 blocks of presentations (150 trials in all), on average, only a few KCs developed sensitivity to a given odor leading to a sparse representation for that odor. This was mainly a result of the odor specific decrease in synaptic weights from AL to MB during learning with repeated odor trials. After learning, only few cells received a combination of the PN inputs that was sufficient to trigger a postsynaptic spike.

Odor induced sparse responses in KCs usually involved a single spike (e.g., Figure 2E, and Figure 3 middle and right column). Even though all the odors were generated by the same stochastic process, there were a few exceptions: for odors 3 and 8 no KC became specific. Population sparseness was increased in odor 7, without reaching single cell specificity. The lack of specific response in these simulations likely arose because only a small fraction of all PN combinations had real KC targets in our model. Similarly, a single experiment *in vivo* sampling only a fraction of the total KC population may reveal no responses to a given odor [7]. Overall, odor representations were significantly sparser after synaptic tuning through SRDP (*p*<0.0002, paired *t*-test on sparseness measures, see Materials and Methods; Figure 3B) and STDP (*p*<0.0003) compared to the naive case. In general, both SRDP and STDP mechanisms were able to tune the synapses between PNs and KCs such that KC responses to different odors were specific, sparse and reliable across trials.

### Synaptic Tuning

**Figure 4 pcbi-1000062-g004:**
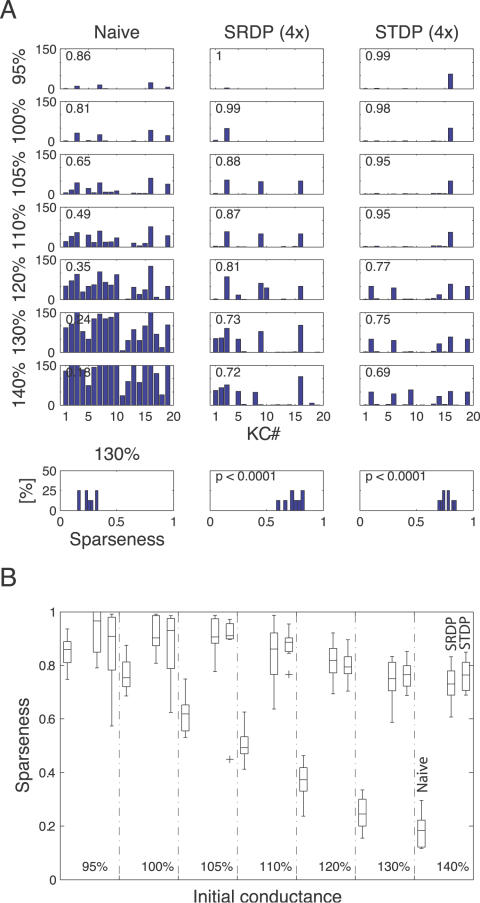
Synaptic tuning over a wide range of initial conductance values. (A) Left column: spike count bar plots for naive population responses (100%: reference value 170 pS; see text). Middle column: responses after SRDP-based synaptic tuning during presentation of 4 blocks of olfactory stimuli. Right column: the same for STDP. The same test odor was used in all conditions. The sparseness measure *Sp* for each condition is indicated in the upper left corner of the bar plots. Bottom row: Histogram of the sparseness measure at 130% of initial conductance value across 8 different odors, in the naive, SRDP, and STDP cases. Note the dramatic increase in sparseness following both synaptic plasticity mechanisms. (B) Sparseness as a function of initial synaptic conductance value in the naive, SRDP, and STDP cases. In each conductance condition the distribution of sparseness values *Sp* across odors is indicated as boxplots. Each box represents the lower quartile, median, and upper quartile. The whiskers extend from each end of the box for 1.5 times the interquartile range. Outliers are indicated as crosses.

We next asked whether plasticity could tune synaptic strengths for a wide range of initial conductance values at KC synapses. Training experiments were performed over 4 blocks of stimuli, starting from different values of initial conductance that were derived from the 100% reference value used for all other experiments (see Materials and Methods). When initial conductance was increased, naive responses became denser: more and more KCs were recruited in the stimulus-induced population firing, and most KCs started to fire repeatedly during a single odor trial (Figure 4A, left column). Training with either SRDP or STDP made the population response much sparser (Figure 4A, mid and right column). Even for initial conductances in the 130–140% range, almost all neurons involved in the response fired a single spike after olfactory experience, whereas more than half of the KC population remained silent for all odors. For example, across all odor samples, the sparseness measure at 130% was significantly different from the naive case for both SRDP (*p*<0.0001; paired *t*-test) and STDP (*p*<0.0001; Figure 4A, bottom row). In general, population sparseness across all odors decreased linearly with increasing initial conductance in the naive case (Figure 4B). Yet both rate- and timing-dependent plasticity mechanisms produced the same near maximal sparseness, with somewhat lower sparseness when initial conductances exceeded 120%. Thus, activity-dependent synaptic tuning is able to normalize postsynaptic neuronal responses over a wide range of initial conditions, and can do so without a global scaling signal [30],[31]. Additionally, this finding strongly mitigates the choice of initial synaptic strengths for computational models incorporating synaptic mechanisms with forms of plasticity analogous to SRDP or STDP: such models require less fine-tuning to work well. Finally, these forms of synaptic plasticity appear to favor a sparser code within a neuron population of fixed size, thereby increasing the global storage capacity of the system [5]. We therefore asked whether the system is able to respond precisely and sparsely to additional odors after being trained by one odor.

### The Persistence of Olfactory Representations

Once generated, an olfactory representation should not only be reliable whenever activated by the same odor (Figure 3), but should also be reproducible after training with other odors. When plasticity is active, representations risk corruption by exposure to new odors and by ongoing activity. We compared the ability of SRDP and STDP to tune synaptic strengths such that multiple odor representations could coexist in a stable manner within a fixed pool of KCs. In doing so, we did not assume that odor memories are stored exclusively at the AL-MB synapses, nor did we aim to fully characterize the degree to which representations are protected against degradation. Rather, we tested whether an activity dependent tuning mechanism is compatible with multiple coexisting representations. Other mechanisms not further explored here may collectively support long-term memory retention [32].

**Figure 5:**
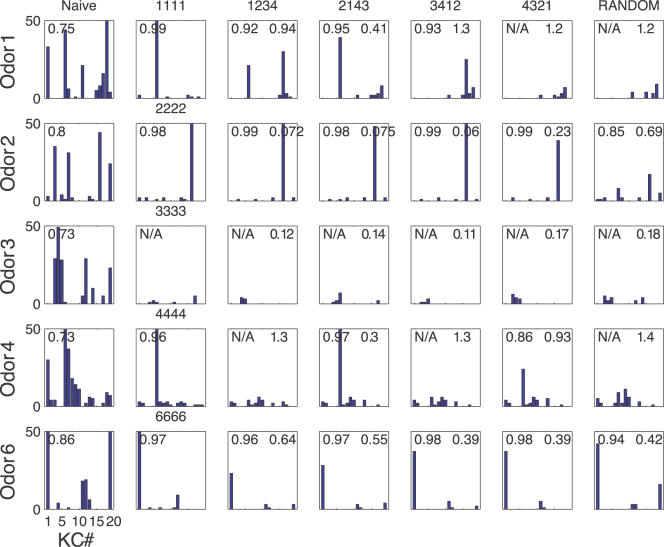
The persistence of olfactory representations. Columns: different response patterns of the KC population with respect to STDP-based plastic exposure to different arrangements of odors 1, 2, 3, and 4. From left to right: “naive”; after learning on a test odor alone (e.g., “1111”: four blocks of odor 1); after sequential learning on combinations of four different odor blocks (e.g., “2143”: presentation of one block of each odor, i.e., odor 2, followed by odor 1, odor 4 and odor 3); “RANDOM”, after learning on a sequence of randomly permuted single 1-s trials of the odors 1, 2, 3, and 4. Rows: the olfactory circuits above were tested by presenting one block of a single odor in the learning sequence. Last row: the same olfactory circuits were additionally tested with the presentation of one block of an odor (odor 6) that was not included in the learning sequence. The sparseness measure *Sp* for each condition is indicated in the upper left corner of the spike count bar plots (N/A: not available, less than 1/4 of 1-s trials elicited on average at least one spike). For each multiple odor sequence, the Euclidean distance in 19-D KC odor space from the average representation after learning on a test odor alone is indicated in the upper right corner.

We trained simulated olfactory circuits on four different odor blocks (odors 1, 2, 3, 4) delivered in various sequences (Figure 5). Each sequence consisted of 200 1-s trials in total, each odor block contributing the same 50 trials to every sequence. The sequences were labeled 1234, 2143, 3412 and 4321, indicating the order in which each odor block was presented. In an additional sequence, the 200 1-s trials were randomly permuted. After the training period, each model circuit was tested as usual on a single odor block, without modifying synaptic strengths.

To quantify the extent to which a given odor representation (e.g., odor 2) acquired during training from a sequence (e.g., 1234) deviated on average from the representation obtained without presentation of other odorants, we computed its Euclidean distance (in KC odor space, see Materials and Methods) from the average response after training with the test odor alone (e.g. 2222). A null distance indicated perfect response matching. For reference, the average pairwise distance between representations obtained after training with a single odor (Figure 3A, 8 odor-specific responses) was 1.3±0.9 for SRDP and 2.7±1.7 for STDP.

After SRDP- or STDP–based training with the multiple odor sequences, the observed KC population responses to single odors were significantly more sparse than in the naive case, and comparable across different sequences (SRDP: *F* = 12.66, *df* = 12, *p*<0.0015; STDP: *F* = 15.12, *df* = 17, *p*<0.0001; 1-way analyses of variance).

Olfactory representations for single odors acquired during training with STDP on multiple sequences had firing patterns that were very similar to those observed after learning each odor alone (Figure 5). The random sequence was less effective at producing reliable odor representation, indicating that repeated consecutive presentations of 1-s trials from the same odor are needed to form a more stable representation. Exposure to odors not included in the training sequences (e.g. odors 5, 6, 7, 8, in Figure 3) in one case reproduced the expected pattern with remarkable similarity (odor 6, Figure 5). For the other odors, the responses after olfactory experience remained similar to their respective naive case (not shown). These results suggest that training by a set of odors presented even in random sequences could tune the system and lead to high KCs specificity for each of them. What is important is an extensive “coverage” of the odor space by the set of odors used for training. Thus, training by a set of similar odors can leave some of AL to MB afferents unchanged, so the following presentation of another “different” odor may lead to a non-sparse response. In contrast, a set of more distinct odors would tune the whole system for all possible inputs including those not used for training. This “tuning” with even an impoverished set of odorants could occur at early stages of development. This result suggests some behavioral consequences, e.g., animals growing in a very stable and poor odor environment should show poor performance in discriminating among similar odors if these odors belong to a novel (for that animal) chemical group.

We found that the rate-based learning rule was less successful in storing multiple odors. Exposure to a single odor after olfactory experience with multiple odor sequences with SRDP induced firing patterns with mixed degrees of similarity to those expected and observed after training with a single odor (Figure S1). The expected pattern was reproduced only in 44% of the tested samples. The remaining combinations either yielded “wrong” patterns (13%) or did not elicit any reliable response. In some cases, odor sequence training induced a KC to become specific to an odor to which it was not responding when trained on the same odor alone (e.g., sequence 1234 tested with odor 2; Figure S1). Interestingly, the best reproductions of the expected, single odor-trained olfactory representation were observed when presenting the first odor of each training sequence, e.g. odor 1 for 1234, odor 2 for 2143, etc., indicating that under SRDP the first odor in the sequence had a major effect on the synaptic strengths of KCs. Also in the SRDP case, the random sequence did not induce any significant persistent odor representation.

Finally, exposure under SRDP to odors not included in the training sequences did not reproduce the original patterns, and responses were often characterized by widespread silence. Thus, although the two learning rules performed similarly in the previous experiments, the results with odor sequences indicate that synaptic changes induced by STDP are more specific and less disruptive than SRDP, allowing persistent coexistence of multiple olfactory representations. This result can be explained in part by the more significant alternations of synaptic weights observed with SRDP model (see below). Note that the performance difference between the two plasticity models cannot be equalized by modifying the model parameters to reduce synaptic changes associated with SRDP. Such a change would actually decrease the SRDP model performance in experiments with single odor presentations.

It is important to emphasize a difference between olfactory stimuli and other modes of sensory input, e.g., visual. In our model, each odor stimulus could be considered a sequence of independent pulses or bins, each bin corresponding to one cycle of LFP oscillations. Each cycle consisted of a unique combination of active PNs that were synchronized to each other, and all together provided an input that may be sufficient to induce spiking in a postsynaptic KC. Only small subset of all possible 10 cell combinations of PNs had a “real” target (one of 19 KCs) in our model. Each KC produced typically zero or one spike during the whole 1 sec odor stimulus, responding to a unique combination of synchronized PNs found at one of many LFP cycles. Coincidence detection properties of the KCs in our model prevented integration across many cycles making each cycle essentially independent. In other words, each unique pattern of PN activity at each LFP cycle during 1 sec odor presentation could be treated as a separate stimulus – brief odor pulses. Therefore, one possible interpretation of the above experiments on representation persistence is that a set of 20 different stimuli presented as a sequence (first 1 sec odor) was used to train a model circuit, followed by application of 3x20 (2d, 3d and 4th odors) independent stimuli. Finally, the representation persistence was tested using the original set of 20 stimuli. Results of simulations support a coexistence of multiple odor representations; the sparseness of KC responses guaranteed little interaction between different odor representations.

### Synaptic Strength Dynamics

**Figure 6:**
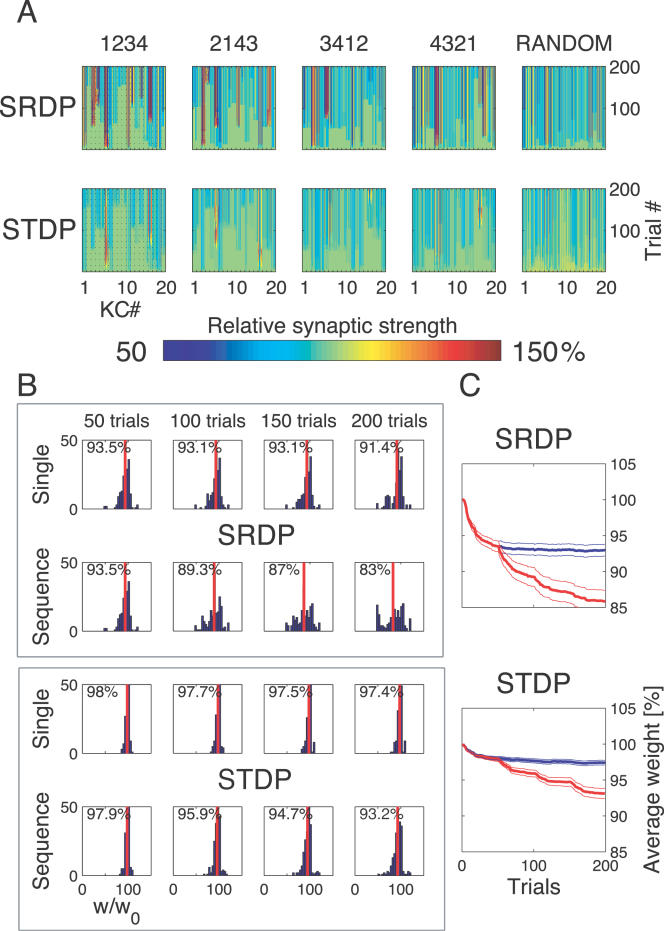
Synaptic strength dynamics. (A) Evolution of synaptic strength during presentation of odor sequences (columns) as in Figure 5. Top row: SRDP; bottom row: STDP. In every diagram, the strength of each PN-KC synapse is color coded, from bottom to top, across the 200 1-s learning trials (vertical axis). The corresponding KC is indicated on the horizontal axis, 10 synapses per KC (dotted grid). Values normalized to the initial conductance g* and represented color coded as percentage of change. Note how STDP induces more selective and less dramatic changes. (B) Distribution of synaptic strengths during learning after 50, 100, 150, and 200 learning trials (columns), as in Figure 5. Top box: SRDP; bottom box: STDP. Each box compares values for single odor learning (upper row) with values for learning odor sequences (lower row). Single odor distributions include the average synaptic values for the four odors 1, 2, 3, and 4, whereas sequence distributions include the average synaptic values for the odor sequences “1234,” “2143,” “3421,” and “4321.” Normalization as in (A). Vertical red lines indicate mean values. (C) Average (±SEM) synaptic value during learning of single odors (blue) or odor sequences (red). Values and normalization as in (A).

To better understand the mechanisms underlying the formation of the response patterns to multiple odor sequences, we analyzed the evolution of the synaptic strengths under each learning mechanism. While training with odor sequences, changes under SRDP affected most KCs, and most synapses at each KC (Figure 6A, upper row). The proportion of synapses undergoing strong changes was larger than for STDP, as shown by the wider and higher kurtotic distributions of synaptic efficacies for increasing number of training trials and learned odors (Figure 6B, upper rows). In contrast, synaptic changes under the STDP rule were subtler, and restricted to fewer KCs and fewer synapses (Figure 6A and 6B). As expected from the specified dependence on instantaneous conductance (Figure 1D), both rules generated unimodal distributions of synaptic efficacies, for both single odor and odor sequence olfactory experiences (Figure 6B). The average evolution of synaptic strength shows that SRDP had a stronger impact on KC synapses (Figure 6C). After SRDP learning of the first odor in a sequence (or 50 single odor learning trials), the average change was comparable to the value reached with STDP after exposure to the full 4-odor sequence (200 trials, Figure 6C). Thus, under these conditions, STDP is more selective than SRDP. This selectivity should allow for a larger number of odors to be learned and represented with a fixed number of synapses.

### Sparse Representations for Similar Odors

In honeybees, it has been demonstrated that selective disruption of oscillatory synchronization by picrotoxin injections into the AL impaired the discrimination of molecularly similar odors [12]. If oscillatory synchronization of neuronal assemblies is essential for fine sensory discrimination, and MB decoding in part depends on the identity of active KCs, then KCs must be able to build distinct sparse representations for similar odors based on differences in stimulus-evoked oscillatory synchronization of the PN neural assemblies. We investigated how STDP in the MB might help to shape distinct sparse representations for odorants with similar encoding in the AL (see Materials and Methods for a description of similar and different odors).

**Figure 7:**
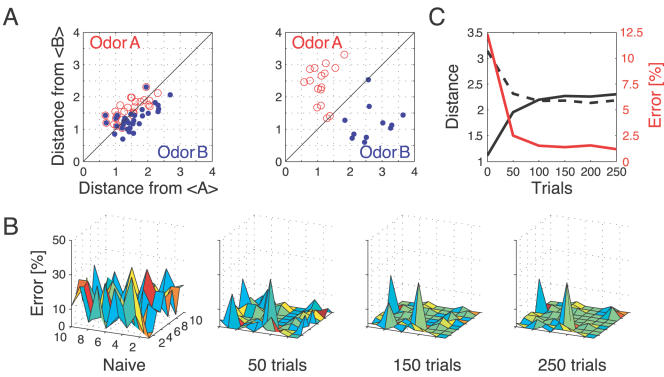
Discrimination of similar odors in 19-dimensional KC space. (A) Clustering of responses to two similar odors A and B (*P_S_* = 0.02) for a naive system (left panel) and after STDP-based synaptic tuning (right panel). Representations for 1-s odor trials of each odor (dots) in KC space were compared by computing the Euclidean distance from the average response to odor A (vertical axis), and the average response to odor B (horizontal axis). Diagonal line delineates classification. For this odor pair, classification was perfect after learning. (B) Wrongly classified trials (error) between pairwise comparisons of 10 similar odors at different stages of learning. (C) The substantial average drop in classification error rate across all odor pairs in (B) (red, right axis) can be ascribed to a matching increase of the distance between average odor responses (“cloud centers” in KC space; black, left axis) and a decrease in “cloud size” (twice the average distance of single responses to one odor from their respective “center”; dashed black line, left axis).

First, we tested the model's ability to discriminate pairs of very similar odors extracted from a set of 10 odors (*P_S_*≈0, see Materials and Methods; Figure 7). We defined an odor trial as correctly classified if its representation in KC odor space was, with cross-validation, closer to its corresponding average response than to any other average odor response. The classification error was defined as the percentage of wrongly classified trials in a block of 50 trials. Olfactory representations for two similar odors A and B were largely overlapping in naive circuitry (Figure 7A, left), and many responses to odor A were closer to the average response to B. After synaptic tuning, all single-trial responses to odor A were closer to its own corresponding response average (Figure 7A, right), leading to perfect odor classification. This indicates that the strong increase in KC sensitivity to the fine synchrony structure induced by plasticity effectively improves discrimination of similar odors.

To track the evolution of this enhancement for different amounts of training, we quantified the portion of wrongly classified trials across all possible odor pairs from the set of 10 very similar odors (Figure 7B). Distinct olfactory representations were already obtained with one training block. With two training blocks (100 trials), odor classification improved further, resulting in only three cases of partial overlap. The average evolution of the classification error rate across all odor pairs suggests that an asymptotically small (∼2%) number of classification errors was reached after 100 trials (Figure 7C). We noted that, corresponding to this saturation point, the divergence of average odor responses (cloud centers) in KC odor space also reached an asymptotic value (Figure 7C). After 100 training trials the distance between two cloud centers was always larger than twice the average distance of single responses from their respective centers (cloud radius), explaining the optimal classification results.

**Figure 8:**
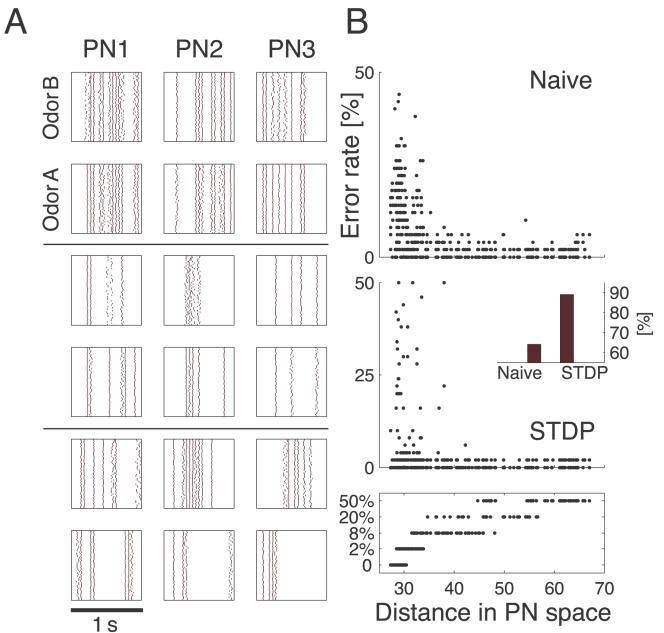
Discrimination in MB versus intrinsic distance in AL. (A) Three odor pairs with identical (upper rows) or different (middle rows: 20% different; bottom rows: totally different) slow temporal structure in AL representation (three cells; spike rasters for 50 trials). Note how the spiking pattern of each PN becomes more and more different when divergence in PN space increases between the two odors. (B) Discrimination error in KC space for a naive system (top panel) and after learning with STDP (middle panel). In each panel a dot represents an odor pair (as in [A]) extracted from one of five sets of 10 odorants (bottom panel) with increasing degree of slow structure dissimilarity in PN space (abscissa) for pairs within the set. For each pair the classification error in KC space is computed as in Figure 7A. Note how plasticity particularly improves classification for odor pairs that have a very similar slow structure representation in PN space. Insert: Global enhancement of discrimination capacity after learning indicated as the proportion of odor pairs with classification error lower than 5%.

To what extent does plasticity-based improvement of KC decoding abilities depend on the difference between PN responses elicited by odors, that is, their mutual distance in PN space? To explore those features over a homogeneous distribution of pairwise distance values, we generated several sets of 10 odorants with increasing degree of dissimilarity between odors within sets (see Materials and Methods; Figure 8A). The closest odors were characterized by very similar (identical for *P_S_* = 0; Figure 8A, upper rows) slow temporal structure but different patterns of PN synchronization (fine structure). For each odor pair in a set, we quantified the distance (or divergence, see Materials and Methods) in PN space (Figure 8B, bottom panel) and classification error in KC space (as above) between the two representations. Before synaptic tuning with STDP (Figure 8B, upper panel), odor pairs with a high degree of similarity in slow structure gave a wide range of error rates (0–45%), suggesting that the naive circuitry is unable to exploit synchrony information to create distinct representations. The fraction of pairs with poor classification was very high, particularly over the range of PN distances covered by the two sets with very similar odors. For larger PN distances, the error rate was nearly constant and mostly below 5%. Thus the classification rate was influenced by differences already expressed in, and transmitted from the AL. After olfactory experience, the error rate for most odor pairs plummeted to below 5% over the whole PN distance range (Figure 8B, mid panel). This suggests that the strong enhancement of classification ability for similar odors (Figure 8B, insert) was provided by the increased sensitivity to oscillatory synchronization patterns after STDP, as expected [12]. Only a small discrete set of odor pairs very close in PN space continued to elicit high rates of classification error. Taken together these results indicate that fine tuning for ensemble selection through a timing-dependent plasticity mechanism in the MB may increase the ability to discriminate between two odorants (Figure 8B insert).

## Discussion

We used a model of the locust olfactory system to show that a form of plasticity acting at the synapses between PN axon collaterals and KC dendrites is an efficient tuning mechanism for MB neurons to respond in a sparse and odor-specific manner to dense odor evoked oscillatory input.

### Synaptic Plasticity and Sparse Representations

Activity-dependent modulation of relevant synapses made population responses in the MB highly selective and odor specific, and increased trial-to-trial reliability, to the extent observed experimentally in the locust olfactory system [7],[33]. Activity-dependent cortical plasticity is not unusual in primary sensory systems, where it contributes to organizing the representation of sensory information in well-ordered maps during development. It has been examined extensively with experiments and models in the visual [24],[26],[34], auditory [22],[35], and somatosensory cortices [36],[37].

What mechanisms enable sparse and reliable KC firing only when they detect the right input combination? In our computational model, plasticity could optimally tune KCs to become selective coincidence detectors of preferred simultaneous PN input. Physiological results from locust MB indicate that coincidence detection results from the non-linear membrane integrating properties of active depolarizing conductances (most likely voltage-dependent) in KCs together with AL driven feed-forward inhibition through lateral horn interneurons (LHIs) [7],[8]. Even with the intrinsic (active conductances) and synaptic (input from LHIs) mechanisms present, the specificity of KCs responses to dynamic input is likely to require “initial” tuning of the synaptic strengths between AL and MB. Indeed, with excessively strong synaptic afferents, as in the case of high initial synaptic conductances (Figure 4), a few aligned PN spikes would be sufficient to induce spiking in a KC. Reducing the KC integration window would reduce the probability of firing; however, the sparseness of the response would still depend on the synaptic weights. Increasing the initial synaptic coupling from AL to MB would sacrifice sparseness. In this situation the KC could not detect stimulus specific synchrony in the input from the AL, resulting in a surplus of KCs firing for each odor presentation. Computational work has shown that only when temporal aspects of coding were not addressed, plasticity in the MB could be omitted [38]. Thus, in our model, synaptic plasticity provides a mechanism for tuning synaptic weights to the MB to enable coincidence detection-based odor encoding by KCs, and to ensure a sparse odor representation in the MB as observed experimentally. However, these results do not imply that odor encoding in the mushroom body depends solely on previous odor experience; synchronous spiking in odor specific populations of PNs controls the KCs response patterns during odor stimulation. Therefore, in the system implementing AL-to-MB plasticity proposed here, the similarity of glomerular olfactory maps and the structure of the olfactory system across individuals (e.g.,[ 39],[40]) can still be reflected in odor-evoked activity in the MB as observed, for example, in *Drosophila* [41].

Here we propose a form of experience-dependent plasticity as a complementary mechanism to stabilize the interface between the two circuits, increasing reliability, providing persistence and maximizing the selectivity of sparse responses to time-varying input. We showed this tuning to be especially useful in situations where precise comparisons between input patterns are required (as when similar odors must be distinguished). Analogous scenarios may occur in other neural systems. In the developing visual system, the fundamental structure of cortical maps appears to be intrinsic [42], yet experience is crucial for maintaining the responsiveness and selectivity of cortical neurons, as well as for defining detailed map features [34]. Other sensory circuits employing coincidence detection have been shown to require plasticity-controlled synaptic tuning to adjust for transmission delays along axonal pathways of variable length, accurately tuning spike arrival times [22]. Here we contend that in the locust all these properties can coexist in the same olfactory circuit and that their combined action on synchronized oscillatory activity contributes to the observed functional transformation of neural codes.

In our model a relatively small fraction of total PN population was connected to each KC. Recent results indicate that a much larger fraction of the PN population (up to 50% of the all PNs in the locust AL) may constitute the “receptor field” of a single KC [43]. With our model we found that changing the fraction of PNs synapsing upon a single KC made little difference to the results as long as all initial synaptic weights were scaled accordingly. Indeed, the fraction of PNs connected to a single KC, the size of the KC integration window and the absolute synaptic weights between AL and MB constitute complimentary parameters that control the sparseness of MB responses. The effect of increasing the number of PNs projecting to a single KC is similar to that of increasing synaptic weights or increasing the integration window in KCs – all lead to a loss of sparseness (see, e.g., Figure 4). Therefore, our study provides a general prediction for how a loss of sparseness in the MB can be recovered by active plasticity mechanisms operating between AL and MB.

Sparseness of KCs responses also depends on the input they receive from LHIs; this rhythmic inhibitory input resets KCs at the end of each oscillatory cycle, thus preventing the integration of spikes from PNs over longer time intervals [7],[8]. And, recently, it was proposed that LHIs maintain the sparseness of KCs' responses across a wide range of odor concentrations by changing the size of the KCs' integration window [44]. Under the conditions considered by our model, however, the duration of the KCs integration window was already limited by the intrinsic properties of KCs (see Materials and Methods; Figure 1F and 1G). Furthermore, in contrast to the case of a change in odor concentration, a change in the synaptic weights between the AL and the MB does not affect the timing of the LHI firing. Therefore, an increase in synaptic weights to the MB would lead to immediate increases in response probability of KCs, which cannot be compensated by LHI input; under these conditions, other mechanisms involving direct alternations of synaptic weights are required to control sparseness.

Between zero (no specific response in any KC) and ∼15% of all KCs, depending upon the odor, remained active after training in our model; a given odor usually induced responses in 5-10% of all KCs. This fraction is somewhat larger than the estimations from the experimental data, likely the consequence of our simulation of odors as the activation of all PN afferents. *In vivo*, an odor would induce measurable responses in only a fraction (∼40%) of the total PN population [45]. Therefore, our results provide an upper estimate to the probability of KC firing.

### Spike-Timing Dependent Plasticity

By selectively strengthening the synapses that participate in the generation of an action potential within the potentiation time window Δt>0 (Figure 1C), STDP triggers competition among the inputs to a neuron [30]. Repeatedly correlated inputs on the timescale of the LTP time window will eventually control postsynaptic firing, whereas less effective, uncorrelated inputs will be weakened. Thus the STDP mechanism was more effective than SRDP at tuning postsynaptic neurons to select synchronized ensembles. Synaptic changes under STDP were in general less intense and more selective (Figure 6), such that a fixed number of synapses could learn to represent a larger number of odors (Figure 5). This was mediated by the fine timing-dependent interplay of LTP and LTD that allows activity-dependent bidirectional modification [46], a computationally useful feature. In addition, acting locally at each synapse, STDP conveyed global stability and normalized synaptic strength [30],[31] (Figure 4), without requiring a global scaling signal [27].

Why does STDP favor sparse representations in the MB? A spike occurred in a KC only when input from the AL exceeded a predefined threshold. We found that vigorous spiking occurred throughout the MB when this threshold was set low – then, many KCs received input sufficient to trigger a spike. Because different KCs sample different sets of PNs, dense spiking can only occur when synapses are relatively strong, permitting only few input spikes to depolarize KCs above the spike threshold. Thus, there is a strong relationship between the sparseness of responses in KCs and synaptic weights from AL to MB. This relationship also underlies our observation that, when MB responses are dense, only the first few of a series of spikes from PNs received by KCs may have positive STDP timing; the rest of the spikes will arrive after the KC has fired an action potential, causing the respective synapses to be weakened by STDP. Thus, for each KC, plasticity acts to facilitate only a few of the input synapses and depresses the others. The overall effect of this dynamic is a decrease in net synaptic weight (Figure 6). Synaptic depression halts only when synaptic weights become sufficiently weak that the collective action of the majority of the input spikes is required to trigger a postsynaptic KC response. Because only a fraction of the PNs is synchronized during an odor response, only a few KCs may receive enough synchronized input to spike. So, the process converges to the state that is characterized by sparse odor representation in the MB.

Whereas the functional significance of oscillatory synchronization in neural circuits is generally uncertain, in the insect AL it has been shown to be essential for fine perceptual discrimination [12]. Our model replicated this aspect of coding, as shown by simulations with PN firing patterns with the same slow temporal structure but different cycle-specific PN synchronization. Because of the intrinsic sensitivity to such synchronous ensembles, STDP tuning efficiently enhanced the ability of the circuit to discriminate among similar odors (simulated by similar PN response patterns), significantly reducing classification errors. In general, STDP was more effective than SRDP, which has implications for future experimental investigations of plasticity mechanisms in insects MB. We cannot exclude the possibility that, for different types of neuronal networks and different input patterns, SRDP may prove as efficient as STDP did in our model of the locust olfactory system. Particularly, models of sensory processing which do not depend on the synchrony of presynaptic spikes may benefit less from STDP. We tested a wide range of parameters in our model and always found STDP to perform better than SRDP. Interestingly, STDP was recently observed to occur at the downstream (projected to the alpha/beta lobes) synapses from KCs [47]. This finding suggests that forms of plasticity mediated by spike timing are not unique to vertebrates, and provides support for our predictions regarding a role for STDP in fine tuning the synaptic weights between AL and MB.

### Plasticity in the MB of Insects

Synaptic plasticity in the MB dendrites is a mechanism that may subserve different functions. In *Drosophila*, synaptic transmission between KC and downstream populations is necessary for memory retrieval but not for memory acquisition or consolidation [19],[20]. This suggests that synaptic connections from AL to KC dendrites may be the sites of functional plasticity relevant to the acquisition and storage of memories. Our results strengthen this idea, showing that a circuit trained with several different stimuli can retain response specificity (Figure 5). However, the main prediction of our study is that synaptic plasticity on the KCs dendrites is important to ensure optimal (sparse) MB responses after sufficient odor experience. It suggests that a broad enough set of distinct odors could tune the whole system and ensure optimal MB responses for all possible inputs including those not used for training. Thus, our study is consistent with a recent set of experimental results supporting a pre-synaptic mechanism of plasticity between Kenyon cells and mushroom body extrinsic neurons as a critical process in olfactory memory [48]–[50]. Finally, recent intracellular recordings made in vivo in locust revealed that individual synapses from KCs onto downstream targets are excitatory and undergo STDP on a +/−25 ms timescale [47]. This finding not only confirms the plausibility of STDP mechanisms in the locust olfactory circuitry, but also shows that these mechanisms appear to have properties similar to those found in vertebrates, directly supporting our strategy of selecting learning rule mechanisms and parameters.

What are the mechanisms underlying changes in synaptic strength in insects? There is growing evidence that plasticity mechanisms acting on different time scales are widespread in the olfactory circuit of locusts and other invertebrates. In honeybee, experience- and age-related changes in KC dendritic trees have been reported [51]. In cricket, dendritic spines of KCs exhibited high concentrations of the protein f-actin, suggesting that the calyx may contain sites of synaptic and structural plasticity [52]. More importantly, activity-dependent modifications resembling fast learning have been observed in locust AL [6], where stimulus specific-responses of PN assemblies rapidly decreased in intensity, while increasing in spike time precision and cross-neuronal oscillatory coherence. Yet, and most importantly, the recent experimental observation of STDP at excitatory synapses directly downstream of the locust MB [47] implies that mechanisms implementing coincidence detection do exists in this insect. Finally, evidence from *Drosophila* suggests that alternative mechanisms based on the cAMP signaling cascade might convey similar features [53],[54].

It will be important and interesting to examine the results of our theoretical work with experiments performed *in vivo*. Recent work [47] has demonstrated that, in the locust olfactory pathway, STPD helps maintain timing precision at the synapse connecting KCs and their follower neurons in the beta lobe. The direct measurements of PN-to-KC synaptic strength needed to test our model results in vivo will be challenging, because individual synapses are very weak [43]. However, studies pairing direct stimulation of the PN output pathway with odor presentations (which cyclically depolarize KCs) should enable tests of timing hypotheses posed here.

### Synaptic Plasticity and the Neural Code

There is a consensus, supported by a large body of experimental evidence [34]–[37], that activity-dependent synaptic plasticity is the general mechanism underlying the formation of memory representations in the brain during learning and development. However, understanding these processes at the systems level requires an integrated study of elemental synaptic mechanisms with neural coding strategies at the level of neuronal ensembles, as well as their complex interaction within neural networks. In the cerebral cortex and other major systems, the spatio-temporal structure of the input to neuronal populations is largely unknown, making it difficult to assess the role of plasticity rules such as STDP in information processing and memory formation. In contrast, in the insect olfactory system much is known about the intrinsic circuitry [7],[40], functional architecture [6],[12], functional properties of neuronal ensembles [3],[28],[33] and their combined role in odor perception. Because the spatio-temporal structure of the input to the MB is well characterized [3],[9],[10],[28],[33], the locust is an ideal system to study the interaction of neural coding strategies with plasticity and learning mechanisms.

In particular, our results suggest a role for plasticity in the synthesis of information whenever oscillatory neuronal ensembles are revealed by LFP or EEG oscillations. At the synapses along the dendritic tree of a cortical neuron, STDP may, for example, favor the exclusive selection of the combined output of an upstream ensemble labeled as belonging together, and differentiated from other assemblies, by the transient synchronization of individual spikes [1],[3],[10],[15]. Although these predictions are biologically plausible, they may be challenging to test. Depending on input firing rate and the duration of transient synchronization, the neural decoder could respond vigorously or, as in locust, sparsely [7]. In larger systems such as mammalian cortex, sparse activity could be difficult to detect.

In summary, we used a computational approach to examine together the separate mechanisms contributing to olfactory perceptual learning. Population activity in the AL reformats and represents every sensory attribute of the stimuli, constructing a “neuronal version” of the olfactory world. The MB, as a specialized “read-out” circuit, extracts information from that representation for further processing and memory formation. We found that an activity-dependent plasticity mechanism operating at the same synapses involved with the read-out can maximize and stabilize the transfer of specific sensory information between the two structures, ensuring a consistent decoding.

## Materials and Methods

### Modeling Neurons and Neural Circuits

The neural circuit models were implemented with biologically realistic neurons using NEURON [55], a simulation environment for empirically based neuronal modeling. KCs were modeled as single-compartment cells with fast Hodgkin-Huxley-type spiking dynamics:
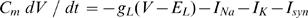
(2)where *C_m_* is the membrane capacitance, *g_L_* is the leakage conductance, *E_L_* is the reversal potential, *I_Na_* and *I_K_* are active Hodgkin-Huxley-type intrinsic currents, and *I_syn_* is a sum of synaptic currents [56]. Data on the biophysical properties of locust KCs are not yet available. Therefore, we approximated KC properties with general descriptions borrowed from other cell types. We were guided by two principles: (1) minimize the number and complexity of ionic currents; (2) generate realistic (though simplified) firing profiles. Previous modeling studies based on a similar methodology revealed the cellular and network mechanisms responsible for the patterning of odor responses in the locust AL [9],[10].

In the model, the width of the timing window for detection of coincident inputs resulted from biophysical properties (internal parameters) of the KC. We found that, for a naive KC (i.e., before plasticity), at least 8 afferent spikes were required to induce firing; when equally spaced, these could maximally span an 8-ms window. The coincidence detection features of a naive KC were systematically tested by generating many combinations of 10 input spikes with timing distributed according to Gaussian functions of increasing variance (Figure 1F). These combinations differed in the interval between first and last spike (window range; Figure 1G). For equal ranges, the combinations had different sequences of spikes within the window. We observed that coincident spikes reliably elicited KC firing for any input combination occurring within a 20 ms window. For larger windows detection reliability decreased (30% at 30 ms) and almost vanished above 40 ms. In our model we omitted feedforward inhibition from lateral horn interneurons, a pathway that has been shown to constrain the coincidence detection window to about 25 ms [7]. This allowed us to assess the effects of synaptic plasticity on response selectivity and sparseness separately from other timing constraints.

### Spike-Rate Dependent Plasticity

Synaptic modification for SRDP was determined according to a modified covariance learning rule [25] that was applied at each PN-KC synapse every 100 ms (i.e. 10 times during a 1-s presentation trial, or every 2nd oscillation cycle; Figure 1A). For each test frame, the time of each KC spike was identified, and the number of spikes for each input PN in the preceding 100 ms determined the PN instantaneous firing rate. When a KC spike was preceded by two or more spikes in one PN, the corresponding synaptic conductance would be increased; one or no PN spike would lead to conductance decrease (Figure 1B). Because KC spontaneous firing rate is extremely low (median 0.025 spike/sec; [7]), the KC covariance term was replaced by a binary element conditioned on KC firing (i.e. conductance changes as above, as long as KC fired at least once; see Figure 1B).

### Spike-Timing Dependent Plasticity

For every PN-KC synapse, the timing difference Δt = *t_post_*−*t_pre_* between pre- and post-synaptic spiking events determined the direction and amount of synaptic modification. Characterization of the pairing time window in cultured hippocampal neurons [57] (Figure 1C), showed that for both LTP and LTD the change in synaptic strength decays according to an exponential function *L_r_* ^±^(*Δt*) = *exp(−Δt / τ* ^±^ *)* (see Equation 1). Thus, a presynaptic spike preceding a postsynaptic spike in an interval covered by the critical timing window produced a conductance increase. A presynaptic spike occurring after the postsynaptic spike produced a conductance decrease.

STDP introduces several useful computational features that have been explored by previous modeling studies [24],[30],[31],[58] and have been incorporated here. Thus, it was assumed that every synaptic pairing event could potentially trigger a change of the value of *g*, with the approximation that the successive modifications elicited by matched pairings sum linearly after weight adjustment [24],[30]. We assumed a longer time constant for LTD (*τ* ^−^ = 20 ms) than LTP (*τ* ^+^ = 10 ms) [59] to provide sensitivity to input correlations over much longer timescales than STDP with equal time constants [24] and to ensure overall weakening of synaptic efficacy for random pairings. Motivated by experimental [59] and modeling [60] studies, it was further assumed that each postsynaptic spike interacts with the presynaptic spikes immediately preceding and following it (nearest-spike interactions).

### Dependence on Synaptic Strength

Theoretical work has long suggested that the intrinsic positive feedback instability of Hebbian learning rules requires models to incorporate constraints to avoid the unrestrained escalation of conductance values, to obtain asymptotically stable distributions, and to reproduce plasticity mechanisms like those observed experimentally (reviewed in [61]). The choice of constraints strongly influences the behavior of the model. Imposing hard bounds on synaptic efficacies results in bimodal synaptic strength distributions with values concentrated around the boundaries (e.g.,[22],[23],[30],[31],[58]). This is unrealistic in the MB, where KCs need flexibility to acquire new odors through synapses not involved in the representation of previously learned stimuli. To obtain stable unimodal distributions of synaptic efficacies, we therefore modeled changes in synaptic strength as dependent on the instantaneous conductance value, proposing linear dependence through factor *C_i_* (see Equation 1) [58],[60],[62] (Figure 1D). This is supported by experimental observations indicating that size-dependent potentiation is weaker for stronger synapses [57],[63],[64], whereas synaptic weakening seems to be proportional to synapse strength [57].

Any selected initial conductance value g* was augmented by a 5% change (*C_i_*) after one single pairing (Figure 1D). Under independence between pairing events, this assumption is supported by data from LTP experiments in which changes in synaptic efficacy are quantified over multiple pairing events (e.g., 60 pairings; [57]). For a typical initial conductance value g* of 170 picoSiemens (pS), a 5% change leads to a change of 8.5 pS, in agreement with previous modeling studies (e.g., 7 pS, [60]). Some results suggest that individual potentiation and depression plasticity events are discrete and heterogeneous in nature [65],[66]. It is still unclear, however, whether or not this is true *in vivo*. Since synaptic plasticity is proposed here as a mechanism to adjust AL-MB synapses to provide sparseness of representations (and not as a mechanism for precise memory changes), the main results of this study remain correct as long as synaptic plasticity provides overall weakening of synaptic efficacy for random pairings (see above). Because experimental evidence suggests that cumulative changes in synaptic efficacy larger than 150% of the initial synaptic value are unlikely [57], conductances larger than 150% of the initial value did not further potentiate. The relative amount of synaptic depression was assumed to grow linearly and in proportion to the instantaneous conductance value, with the same absolute rate of change as for potentiation. Hence, any selected g* would initially be reduced by a 10% change.

### Dependence on Pairing Frequency

We further anchored the computational model in physiological data by tying the regulation of synaptic efficacy to the frequency of pairing (factor *F_p_* in Equation 1), as shown experimentally [46]. Specifically, frequency dependence of LTP seems to partially depend on the residual depolarization between action potentials at high (>10 Hz) frequencies [59]. For presynaptic PNs, we adopted reported nominal threshold frequency values of 10 Hz for LTP and 0.1 Hz for LTD [46],[59]. Given the intrinsic low firing rate of KCs, we adjusted their LTP threshold value to 1 Hz (i.e., at least 1 spike per odor trial). PN average firing rate varied over the narrow 10 to 30 Hz range during odor stimulation. Therefore, we did not incorporate frequency-dependent growth and saturation [46], thereby avoiding conflict with the linear summation of the effects of spike pairs.

### Olfactory Stimulation Patterns and Population Codes

Population coding in the AL was modeled as PN spiking activity set to mimic the odor identity encoding observed *in vivo* (Figure 2A). Thus, *in numero*, AL odor responses consisted of spatiotemporal patterns distributed across evolving PN ensembles exhibiting oscillatory synchronization [3],[28] (Figure 2B). Specifically, each 1-s “odor” stimulus was defined by its representation in the AL through a matrix with a unique spatio-temporal pattern of spiking activity across all 100 PNs (Figure 2C, showing a subset of 10 PNs, the input to one KC) [8]. “Different” odors were generated by modeling completely different patterns of PNs activation. In particular, for each odor it was assumed that about 50% of all afferents are active at the first cycle of odor induced oscillations. At the next cycle any active PN (Figure 2C, colored boxes) could then become silent (Figure 2C, white boxes) and vice versa. However, to avoid abrupt changes we assigned higher probability (*P* = 0.65) for any PN to stay in its current state (silent or active) than to change activation state. This procedure created firing patterns for individual PNs with epochs of active (silent) behavior lasting 150–200 msec on average, which was consistent with experimental data [67] and our previous results of AL modeling [9].

Spike timings of active PNs at each trial were calculated from Gaussian distributions with standard deviation σ being function of cell and cycle numbers. Narrow distributions (small σ) characterized cells firing consistently near LFP peak (see below) across trials (synchronized neurons; Figure 2C, pink boxes). Wide distributions characterized neurons spiking randomly from one trial to another (Figure 2C, yellow boxes). For a given neuron, σ changed from one cycle of oscillation to another to model transient patterns of synchronization. To avoid fast switches between synchronous and asynchronous states, we assigned a higher probability for any active PN to preserve its low (or high) σ between cycles. About 50% of all active neurons were synchronized at each cycle of oscillations. This design created a temporal structure where action potentials of each PN were phase-locked with “the LFP” for 1–5 cycles of population oscillations (50–250 ms), and were followed or preceded by epochs of desynchronized firing or silence (Figure 2C), consistent with experimental data [67] and previous modeling results [9],[10].

In summary, on average, about 50% of the PNs were active at each instant of a given odor presentation. About half of those were synchronized with each other, generating 20 Hz oscillations in the population average (referred to as “the LFP”). The identities of both the active PNs and the synchronized subset changed slowly at each 50 ms oscillation cycle over stimulus duration, modeling slow temporal structure and transient spike synchronization, respectively [8]. This pattern was odor specific and preserved across all trials with a given odor. However, precise timing of individual spikes was set to be different for different trials of a given odor, as calculated from Gaussian distributions. The resulting “jitter” of spike timing was centered on the LFP peak: it was small (<10 msec) for synchronized PNs, while it was large (up to 50 msec) for nonsynchronized PNs. This jitter of PN spike timing was an essential source of noise in the input to the MB.

### Olfactory Learning Experiments and Response Quantification

A presentation block of olfactory stimuli consisted in a series of 50 exposures to a 1-s odor trial followed by a 1-s blank period. Either SRDP or STDP mechanisms were engaged during training experiments, which typically involved presentation of multiple blocks. Plasticity mechanisms were disabled during quantifications of population response, which always employed one block (50 trials). Spike raster diagrams illustrating population responses over one presentation block (Figure 2D and 2E, left panels) were summarized by cumulative spike count bar plots (Figure 2D and 2E, right panels; Figures 3–5).

### Population Sparseness

Population sparseness *S_p_* [68] was measured to quantify the extent to which the population code in KCs becomes sparser after repeated exposures to odors:
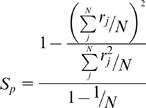
where *N* is the number of units and *r_j_* is the response of unit *j*. This measure of sparseness is inversely proportional to the number of active cells in the population response, varying from 0 for the densest to 1 for the sparsest. To ensure that population sparseness was estimated only for reliable and meaningful responses to a given odor, we required that, at least in one cell, more than 1/4 of the 1-s trials elicited on average at least one spike.

### Odor Space in AL

Odor encoding in the AL was determined by the spike timing structure over all PN-cycle combinations (100 PNs×20 oscillation cycles in a 1-s trial). A special metric was defined to measure the distance (divergence) between two such odor representations. First, for every PN a time series was created by convolving every spike with a Gaussian Kernel (σ = 5 ms, μ = 2σ, full-width-half-maximum = 11.8 ms). The 100 presynaptic spike trains representing the first odor were then compared pairwise with the 100 presynaptic spike trains representing the second odor using an L1-metric (Manhattan distance); this spawned a 100-D vector with the L1-distances. The Euclidean norm of this vector provided the distance measure between two odor representations in PN space.

### Odor Space in MB

Single-trial population output in the MB was examined as a vector in a 19-dimensional (19-D) KC odor space, each dimension counting the spikes generated by one KC during response to a 1-s odor presentation. The average vector over 50 trials represented the average response to a given odor. For illustration, a set of single trial responses can be imagined as a 19-D cloud of points centered on their average response. The Euclidean metric determined the distance (divergence) between two responses. For example, two different odors with ultra sparse representations (i.e., based on one single KC spike per trial) would occupy the unity on orthogonal axes in MB odor space, and therefore their distance would be *sqrt*(2) = 1.44 spikes apart.

### Similar Odorants

Some experiments required sets of similar odorants with a quantifiable degree of within-set similarity. Starting with the AL representation of a master odor (Figure 2B and 2C), a new odor was independently generated by randomly substituting a fraction of PN-cycle spike pattern combinations of the master odor, with a set-specific fixed substitution probability *P_S_*. In other words, the slow temporal structure of odor encoding in AL was altered by *P_S_* • 100%. For each of the 10 new odors in a set, each replacing spike pattern was extracted from the corresponding PN-cycle time window of one of 10 target odors totally different from each other. They were called ‘target’, because with *P_S_* = 1 the slow temporal structure of the master odor would be entirely mutated into that of a target odor. Additionally, the synchrony characteristics (fine structure) of every PN-cycle in a new odor were reassigned independently. Spikes not phase-locked with the LFP could become synchronous, and vice versa. By repeating this procedure with several values of *P_S_* ∈ {0, 0.02, 0.08, 0.2, 0.5}, we were able to control a progressive divergence of the master odor toward the 10 different target odors. Odor pairs in the new sets were thus characterized by varying degree of similarity determined by *P_S_*. All newly generated odors had a comparable ratio of PN-cycle compartments with synchronous spikes, non-synchronous spikes and silence.

### *In Vivo* Recordings

Standard extracellular, “tetrode” recordings were made from PNs in the locust antennal lobe, as described [33].

## Supporting Information

Figure S1 SRDP and the persistence of olfactory representations. Data are represented as in Figure 5. Columns indicate different learning protocols under SRDP. From left to right: “naïve”; learning on a test odor alone (e.g., “1111”); sequential learning on combinations of four different odor blocks (e.g., “2143”); “RANDOM,” learning on the sequence of randomly permuted single 1-s trials of the odors 1, 2, 3, and 4. Rows indicate the test odor for the olfactory circuits above (odors 1–4 were included in the learning sequence; odor 6 was not included in the learning sequence). For each spike count bar plot, the sparseness measure is indicated in the upper left corner (N/A: not available, less than 1/4 of 1-s trials elicited on average at least one spike), whereas the Euclidean distance in 19-D KC odor space from the average representation after learning on a test odor alone is indicated in the upper right corner (multiple odor sequences only).
(0.53 MB EPS)
